# Long Noncoding RNA NEAT1 Regulates TGF-*β*2-Induced Epithelial-Mesenchymal Transition of Lens Epithelial Cells through the miR-34a/Snail1 and miR-204/Zeb1 Pathways

**DOI:** 10.1155/2020/8352579

**Published:** 2020-05-31

**Authors:** Ning Dong

**Affiliations:** Department of Ophthalmology, Beijing Shijitan Hospital, Capital Medical University, Beijing, China

## Abstract

The aim of this study was to explore whether the long noncoding RNA nuclear paraspeckle assembly transcript 1 (NEAT1)/miR-34a/Snail1 and NEAT1/miR-204/Zeb1 pathways are involved in epithelial-mesenchymal transition (EMT) of lens epithelial cells (LECs). Primary human LECs (HLECs) were separated and cultured. Our results identified that TGF-*β*2 induces NEAT1 overexpression in a dose-dependent manner and a time-dependent manner. Additionally, TGF-*β*2 induced downregulation of E-cadherin and upregulation of fibronectin in primary HLECs through a NEAT1-dependent mechanism. Microarray analysis showed that NEAT1 overexpression inhibited the miR-34a and miR-204 levels in the LECs. The expression of miR-34a and miR-204 was decreased, and the levels of Snail1 and Zeb1 were elevated in human posterior capsule opacification- (PCO-) attached LECs and the LECs obtained from anterior subcapsular cataract (ASC) by quantitative RT-PCR (qRT-PCR). Mechanistic studies revealed that NEAT1 negatively regulates miR-34a or miR-204, and miR-34a or miR-204 directly targets Snail1 or Zeb1 by luciferase assay and RNA-binding protein immunoprecipitation assay, respectively. Overall, the NEAT1/miR-34a/Snail1 and NEAT1/miR-204/Zeb1 pathways are involved in TGF-*β*2-induced EMT of HLECs. In summary, TGF-*β*2 induces NEAT1 overexpression, which in turn suggests that NEAT1 acts as a ceRNA targeting Snail1 or Zeb1 by binding miR-34a or miR-204, and promotes the progression of EMT of LECs.

## 1. Introduction

Cataract is a leading cause of visual impairment and blindness globally [1, 2]. It is treatable only by surgical replacement of the cataractous lens fiber mass with an artificial intraocular lens (IOL), which has placed a huge health burden [1, 2]. However, a common complication of cataract surgery is posterior capsule opacification (PCO), which is known as a secondary cataract [3, 4]. PCO is the main cause of vision impairment after cataract surgery, which is mainly caused by proliferation, migration, and epithelial-mesenchymal transition (EMT) of lens epithelial cells (LECs) [3, 4]. Accumulating evidence shows EMT plays an important role in the pathogenesis of PCO [5, 6]. During this transition, residual LECs lose polarity and cell-cell adhesion and transdifferentiate into mesenchyme-like cells [1–6].

TGF-*β* is a requirement for LECs to undergo EMT, which is known as a pivotal inducer of EMT-related changes in PCO [7, 8]. Furthermore, TGF-*β*2 is the predominant isoform in the aqueous humor [9, 10]. Recent research suggests that long noncoding RNAs (lncRNAs) are commonly referred to as non-protein-coding RNA transcripts longer than 200 nucleotides in length [11, 12]. Cumulative evidence reveals that lncRNAs are without functional protein-coding ability; however, they can control several biological processes and play a pivotal role in regulating EMT [11, 12]. Our previous studies have demonstrated that TGF-*β*2 induces overexpression of EMT markers in primary human LECs (HLECs) via a lncRNA metastasis-associated lung adenocarcinoma transcript 1- (MALAT1-) dependent mechanism [6]. The mechanism is that TGF-*β*2 induces MALAT1 overexpression, which in turn suggests that MALAT1 acts as a ceRNA targeting SMAD4 by binding miR-26a, and induces the progression of EMT of LECs [6].

Existing data show a potential contribution of particular lncRNAs to the development of PCO [6, 13, 14]. We have reported that the expression of lncRNA nuclear paraspeckle assembly transcript 1 (NEAT1) is increased by nearly 13-fold in the presence of 5 ng/ml TGF-*β*2 [6]. It is well known that anterior subcapsular cataract (ASC) and PCO share many molecular features such as EMT of LECs [15, 16]. Next, our previous studies have indicated that NEAT1 expression was upregulated by almost 7-fold in human PCO-attached LECs compared with normal-attached LECs and increased by nearly 6-fold in LECs obtained from patients with ASC compared with nuclear cataracts [6].

Nevertheless, whether lncRNA NEAT1 can regulate EMT induced by TGF-*β*2 in HLECs and further contribute to the pathogenesis of PCO is still unknown. Therefore, the aim of the present study was to explore whether TGF-*β*2 induces EMT of primary HLECs via a NEAT1-dependent mechanism.

## 2. Materials and Methods

### 2.1. Patient LEC Collection and Culture

All experiments were approved by the Ethics Committee of Beijing Shijitan Hospital, Capital Medical University (Beijing, China), and performed in accordance with the tenets of the Declaration of Helsinki. Patient LECs were collected and cultured as previously described [5, 6, 17, 18]. Briefly, fresh lens capsules with adherent LECs were obtained from the Department of Ophthalmology, Beijing Shijitan Hospital, during cataract surgery from 66 eyes with the clinical diagnosis of nuclear or anterior polar cataracts. The ages of the patients ranged from 61 to 76 years. Written informed consent was obtained from all subjects prior to their participation in the study. Fresh PCO tissues and normal-attached LEC samples from organ donors were provided by the Eye Bank of Beijing, China (Beijing, China).

Primary HLECs were used to determine the role of lncRNA NEAT1 in EMT of HLECs.

### 2.2. SRA01/04 Cell Culture

SRA01/04 human lens epithelial cells were obtained from the Cancer Institute and Hospital, Chinese Academy of Medical Sciences (Beijing, China). The SRA01/04 cells were cultured as previously described [5, 6, 17, 18]. Briefly, the cells were routinely cultured in Eagle's minimum essential medium (GIBCO BRL, Grand Island, NY, USA) with 10% fetal bovine serum in a 5% CO_2_-humidified atmosphere at 37°C. When the cells were approximately 80% confluent, they were passaged.

The SRA01/04 cell was only used for RNA immunoprecipitation (RIP) study and luciferase assay.

### 2.3. Transfection

Small interfering RNAs (siRNAs) targeting lncRNA NEAT1, including siNEAT1-1 and siNEAT1-2, were obtained from GenePharma Company (Shanghai, China). Primary LECs were transfected with 100 nM siNEAT1-1 or siNEAT1-2 or si-control (negative control siRNA), respectively, for 24 h.  Table 1 shows the siRNA sequences.

In addition, miR-34a mimics, anti-miR-34a, miR-34a mimics control, anti-miR-34a control, miR-204 mimics, anti-miR-204, miR-204 mimics control, and anti-miR-204 control were obtained from GenePharma Company. The primary LECs were transiently transfected with 100 nM miR-34a mimics or anti-miR-34a or miR-204 mimics or anti-miR-204 or negative control for 6 h using GenePORTER transfection reagent (GTS, Inc., San Diego, CA, USA).

To overexpress NEAT1 in the LECs, NEAT1 sequence was cloned into the HindIII and EcoRI sites of the pcDNA3.1 vector (Invitrogen, Carlsbad, CA, USA), named as pcDNA3.1-NEAT1. The empty pCDNA3.1 vector was used as a negative control. The NEAT1 sequence binding miRNA-34a or miRNA-204 response elements were mutated in which 5′-UGUAUAUUUUUGAGGAACUGCCA-3′ changed to 5′-CACCCCCCCCCACAAGGUCAUUC-3′ or 5′-GUUUUCCGAGAACCAAAGGGAG-3′ changed to 5′-ACCCCAAACACCUUCCCAAACA-3′, respectively. The pcDNA3.1-NEAT1 with mutations was named pcDNA3.1-NEAT1-mut (miRNA-34a) or pcDNA3.1-NEAT1-mut (miRNA-204), respectively.

### 2.4. Microarray Analysis

For microarray analysis, the primary HLECs were treated with the pcDNA3.1-NEAT1 or pcDNA3.1-control in 6-well plates for 24 h. Microarray analysis was performed by a Human miRNA Microarray System Version 3 (Agilent Technologies, Santa Clara, CA), which targets differential expression of miRNAs on the primary HLECs treated with the pcDNA3.1-NEAT1 (experiment) and empty pCDNA3.1 vector (control).

### 2.5. Quantitative Reverse Transcription PCR (qRT-PCR)

qRT-PCR was performed as previously described [5, 6, 17, 18]. Briefly, total RNA was isolated from LECs using TRIzol Reagent (Invitrogen, Carlsbad, CA) and reverse-transcribed to cDNA using a PrimeScript RT reagent kit (TaKaRa, Dalian, China). The mRNA levels of the target genes were quantified with SYBR Green-Based Real-Time PCR analysis (Bio-Rad). PCR amplification was performed using a CFX96 Real-Time PCR Detection System (Bio-Rad). Data were analyzed using the comparative threshold cycle (Ct) method, and the results were expressed as the fold difference normalized to GAPDH or U6.  Table 2 shows qRT-PCR primers.

### 2.6. Western Blot Analysis

The primary antibodies, including anti-Snail1 (ab216347, Abcam, Cambridge, MA, USA), anti-Zeb1 (ab203829, Abcam), anti-E-cadherin (ab40772, Abcam), anti-fibronectin (ab45688, Abcam), and anti-actin (ab179467, Abcam), were used for Western blot analysis. The Western blot analysis was performed as previously described [5, 6, 17, 18].

### 2.7. Luciferase Assay

Luciferase assay was performed as previously described [5, 6, 17, 18]. The 3′UTR of Snail1 mRNA and NEAT1 containing the predicted miR-34a binding sites and the 3′UTR of Zeb1 mRNA and NEAT1 containing the predicted miR-204 binding sites or corresponding mutant sites were amplified by PCR. Reporter activities were performed 24 h after transfection using the dual-luciferase reporter assay system (Promega, Madison, WI, USA) [6].

### 2.8. RNA-Binding Protein Immunoprecipitation Assay

RNA immunoprecipitation (RIP) was assessed using an EZ-Magna RIP RNA-binding protein immunoprecipitation kit (Millipore, Billerica, MA, USA) as previously described [6, 19]. An EZ-Magna RIP RNA-binding protein immunoprecipitation kit (Millipore, Billerica, MA, USA) was used for RIP according to the manufacturer's instructions.

### 2.9. Statistical Analysis

All data are presented as the mean ± SE. All statistical analyses were performed using the SPSS for Windows Version 17.0 software (SPSS, Inc., Chicago, IL, USA) [6]. Differences between two independent groups were carried out using Student's *t*-test [6]. Differences among multiple groups were carried out using one-way analysis of variance (ANOVA) and the post hoc test of Tukey's multiple comparisons [6]. *P* values < 0.05 were considered statistically significant.

## 3. Results

### 3.1. TGF-*β*2 Induces Overexpression of EMT Markers in Primary LECs through a NEAT1-Dependent Mechanism

**Figure 1:**
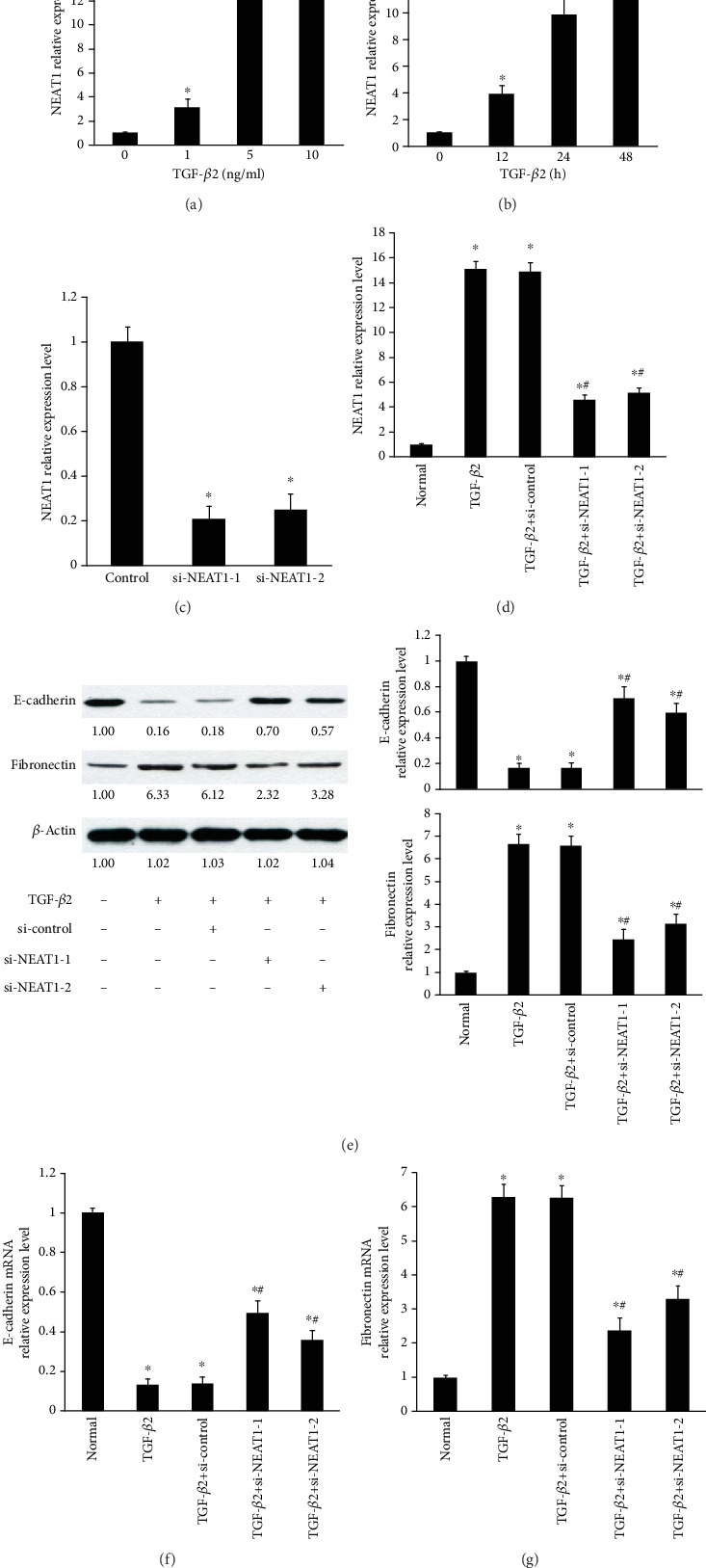
TGF-*β*2 induced overexpression of EMT markers in primary LECs through a NEAT1-dependent mechanism. (a) The primary HLECs were treated with indicated concentration of TGF-*β*2 for 48 h. The expression of NEAT1 was assessed by qRT-PCR. ^∗^*P* < 0.05 compared with the group without TGF-*β*2. (b) The primary HLECs were treated with 5 ng/ml TGF-*β*2 in indicated time. The expression of NEAT1 was assessed by qRT-PCR. ^∗^*P* < 0.05 compared with the group without TGF-*β*2. (c) The expression of NEAT1 was detected by qRT-PCR. ^∗^*P* < 0.05 compared with the control group. (d–g) The primary HLECs were treated with TGF-*β*2 (5 ng/ml) for 48 h before incubation with 100 nM siNEAT1-1 or siNEAT1-2 or si-control for 24 h. ^∗^*P* < 0.05 compared with the normal group. ^#^*P* < 0.05 compared with the group with TGF-*β*2. (d) The expression of NEAT1 was detected by qRT-PCR. (e) The protein levels of E-cadherin and fibronectin were detected by Western blot analysis. (f) E-cadherin mRNA expression was measured by qRT-PCR. (g) Fibronectin mRNA levels were analyzed using qRT-PCR. (a–g) The data are presented as the mean ± SE of six independent experiments.

Our previous studies have identified that NEAT1 expression was upregulated in human PCO-attached LECs compared with normal-attached LECs and increased in LECs obtained from patients with ASC compared with nuclear cataracts [6]. Based on the results, we hypothesized NEAT1 contributes to the pathogenesis of PCO. The previous studies have revealed that TGF-*β*2 is a pivotal inducer of EMT-related changes in PCO [7–10]. Hence, we explored whether TGF-*β*2 induces downregulation of epithelial differentiation markers (i.e., E-cadherin) and upregulation of mesenchymal cell markers (i.e., fibronectin) in primary HLECs through a NEAT1-dependent mechanism. Firstly, the expression of NEAT1 was increased in primary HLECs treated with TGF-*β*2 in a dose-dependent manner and a time-dependent manner (Figures 1(a) and 1(b)). qRT-PCR confirmed the efficiency of NEAT1 knockdown using siNEAT1-1 or siNEAT1-2 (Figure 1(c)). Next, TGF-*β*2 induced the expression of NEAT1 suppressed by NEAT1 knockdown (Figure 1(d)). Besides, TGF-*β*2 induced EMT of primary HLECs, which significantly inhibited the expression of E-cadherin and increased the expression of fibronectin in primary HLECs, detected by Western blot analysis (Figure 1(e)) and qRT-PCR (Figures 1(f) and 1(g)). However, the tendency was reversed by NEAT1 knockdown (Figures 1(e)–1(g)). Immunocytofluorescence further confirmed that TGF-*β*2 induces downregulation of E-cadherin and upregulation of fibronectin in primary HLECs through a NEAT1-dependent mechanism (Supplementary Figure S1).

Overall, these data suggest that NEAT1 contributes to the progression of TGF-*β*2-induced EMT in the LECs.

### 3.2. NEAT1 Regulates miR-34a and miR-204 in Primary HLECs

**Figure 2:**
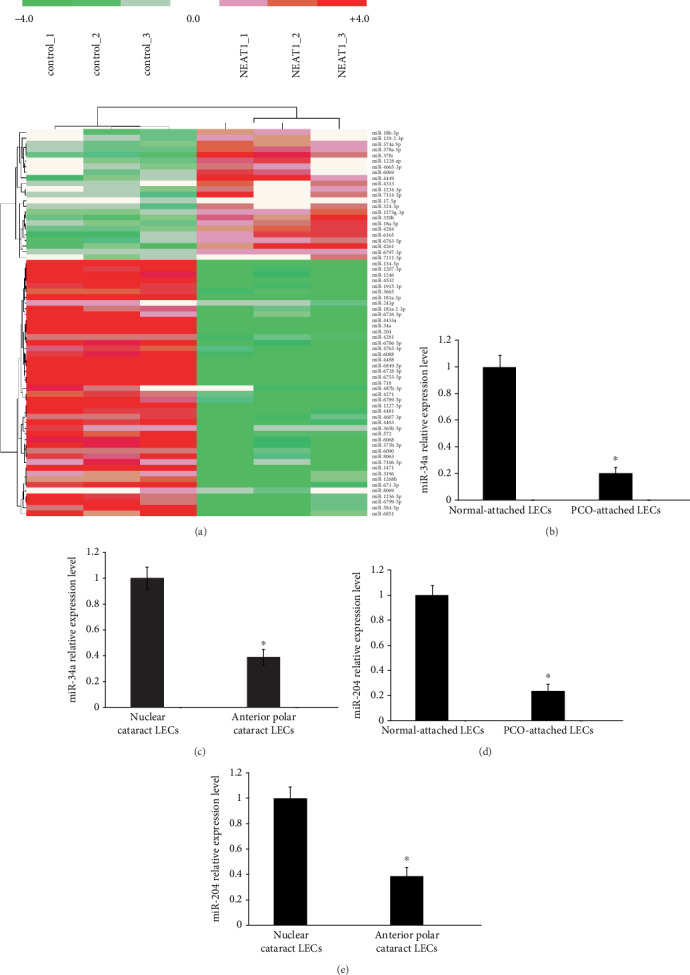
NEAT1 regulates miR-34a and miR-204 in primary LECs. (a) The different expression of miRNAs was shown in the heat map by a Human miRNA Microarray System Version 3. The primary HLECs were treated with the pcDNA3.1-NEAT1 (experiment) and empty pCDNA3.1 vector (control) for 24 h. (b–e) The expression of miR-34a and miR-204 was detected by qRT-PCR. The error bars represent the mean ± SE of six independent experiments. (b, d) ^∗^*P* < 0.05 compared with normal-attached LECs. (c, e) ^∗^*P* < 0.05 compared with nuclear cataracts.

Growing evidence has suggested that miRNA/lncRNA crosstalk by competing endogenous RNAs (ceRNAs) modulates gene expression patterns and controls physiological and pathological processes [20, 21]. To identify the involvement of miRNA/NEAT1 crosstalk in PCO development, we analyzed the different expression of miRNAs in primary HLECs treated with the pcDNA3.1-NEAT1 (experiment) and empty pCDNA3.1 vector (control) using a Human miRNA Microarray System Version 3 (Agilent). The heat map showed that miRNAs were differentially expressed between primary HLECs treated with the pcDNA3.1-NEAT1 and control (Figure 2(a)). A total of 216 miRNAs exhibited significant differential expression (fold change ≥ 2.0, *P* ≤ 0.05) including 110 downregulated miRNAs and 106 upregulated miRNAs. The expression of miR-34a and miR-204 which are the top downregulated miRNAs is decreased by nearly 6-fold in the NEAT1 overexpression HLECs (Figure 2(a)). Next, to confirm the microarray results, the expression of miR-34a and miR-204 was detected in human PCO-attached LECs and normal-attached LECs by qRT-PCR. The expression of miR-34a and miR-204 was downregulated by nearly 5-fold in human PCO-attached LECs (Figures 2(b) and 2(d)). Consistent with the data, miR-34a and miR-204 were significantly decreased by nearly 3-fold in LECs obtained from patients with ASC compared with nuclear cataracts (Figures 2(c) and 2(e)). Previous studies have already demonstrated that miR-34a suppresses proliferation and migration of LECs via downregulation of c-Met and inhibits EMT of LECs by targeting Notch1 [22, 23]. In addition, the previous study has confirmed that miR-204-5p inhibits EMT during human posterior capsule opacification by targeting SMAD4 [24]. Consistent with the previous study, the above results implied that NEAT1 regulates miR-34a and miR-204 in primary HLECs; in addition, miR-34a and miR-204 are involved in the pathogenesis of PCO.

### 3.3. Snail1 Is a Target of miR-34a in Primary HLECs

**Figure 3:**
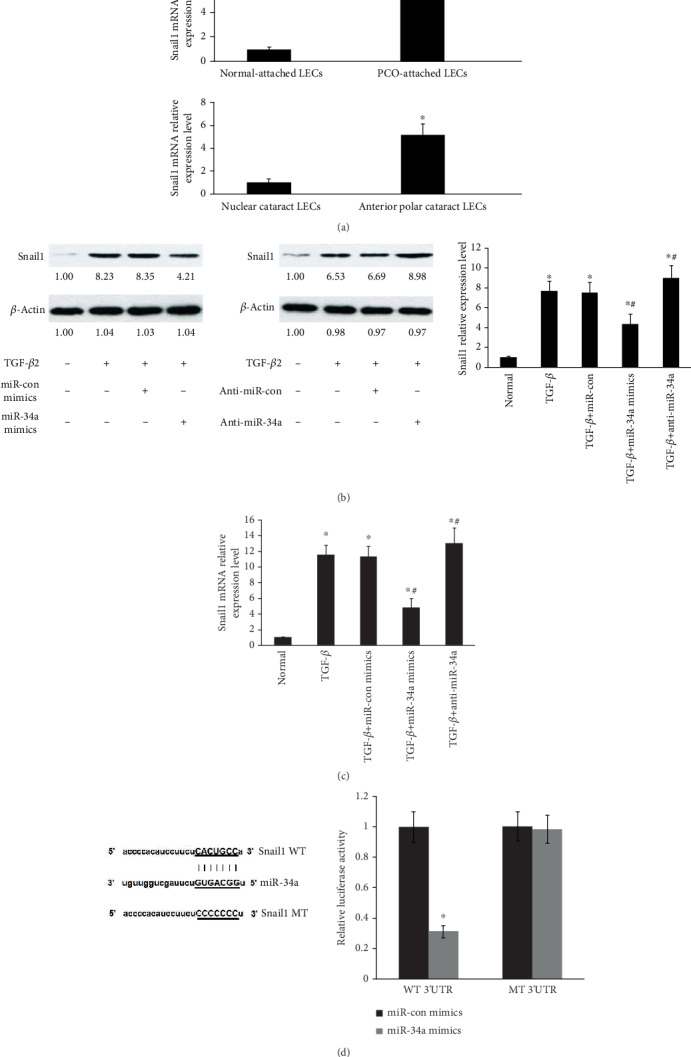
Snail1 is a target of miR-34a in primary HLECs. (a) The mRNA levels of Snail1 were detected by qRT-PCR. ^∗^*P* < 0.05 compared with normal-attached LECs or nuclear cataracts. (b) The levels of Snail1 protein in primary HLECs were determined by Western blot analysis after overexpression or knockdown of miR-34a. (c) The Snail1 mRNA levels were determined by qRT-PCR after overexpression or knockdown of miR-34a. ^∗^*P* < 0.05 compared with the normal or TGF-*β*2 group. (b, c) ^∗^*P* < 0.05 compared with the normal group. ^#^*P* < 0.05 compared with the group with TGF-*β*2. (d) The luciferase reporter assays suggested that miR-34a directly targets Snail1 in LECs. ^∗^*P* < 0.05 compared with the miR-34a control group. The error bars represent the mean ± SE of six independent experiments.

Snail1 (SNAI1) is a transcription factor and typically upregulated induced by TGF-*β* in EMT [25, 26]. The previous studies have indicated that Snail1 contributes to the EMT of LECs [27]. Given that miRNAs can regulate the posttranscriptional expression of protein-coding mRNAs and using TargetScan (http://www.targetscan.org/vert_72/) to search for 3′ untranslated region (UTR) sequences of mRNAs encoding Snail1 [28, 29], we hypothesized that miR-34a can block Snail1 translation through binding to the 3′UTR of it. To confirm these, the levels of Snail1 mRNA were determined by qRT-PCR (Figure 3(a)). Snail1 mRNA was increased by nearly 6-fold in human PCO-attached LECs compared with normal-attached LECs and upregulated by nearly 5-fold in LECs obtained from ASC compared with nuclear cataracts (Figure 3(a)). The data reveal Snail1 is involved in the pathogenesis of PCO. Next, Snail1 induced by TGF-*β*2 was suppressed in miR-34a-overexpressing LECs (Figure 3(b)). Additionally, Snail1 protein expression induced by TGF-*β*2 was elevated in primary LECs treated with anti-miR-34a (Figure 3(b)). Consistent with above data, qRT-PCR showed that Snail1 mRNA induced by TGF-*β*2 was downregulated in miR-34a-overexpressing primary HLECs and upregulated in primary HLECs treated with anti-miR-34a (Figure 3(c)). Finally, the “CACUGCC” which is the 3′UTR of Snail1 mRNA containing the predicted miR-34a binding sites changed into “CCCCCCC”, which was defined as MT 3′UTR. We identified that miR-34a directly targets Snail1 in LECs by luciferase reporter assays (Figure 3(d)).

### 3.4. NEAT1 Negatively Regulated miR-34a Levels

**Figure 4:**
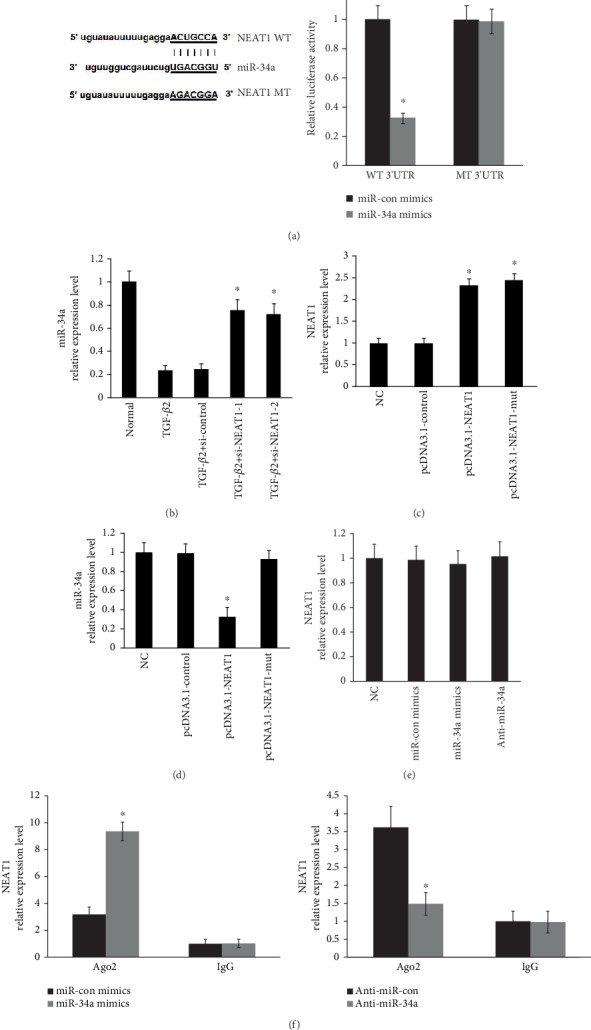
NEAT1 negatively regulated miR-34a levels. (a) NEAT1 contained a binding site for miR-34a in HLECs using the luciferase reporter assays. ^∗^*P* < 0.05 compared with the miR-34a control group. (b) miR-34a levels were determined by qRT-PCR. ^∗^*P* < 0.05 compared with the normal and TGF-*β*2 groups. (c) NEAT1 levels were determined by qRT-PCR. ^∗^*P* < 0.05 compared with the normal and pcDNA3.1-control groups. (d) miR-34a levels were determined by qRT-PCR. ^∗^*P* < 0.05 compared with the pcDNA3.1-NEAT1-mut group. (e) NEAT1 levels were determined by qRT-PCR. (f) NEAT1 levels were determined by qRT-PCR. ^∗^*P* < 0.05 compared with the miRNA control group. The error bars represent the mean ± SE of six independent experiments.

Given that miRNA/lncRNA crosstalk by ceRNAs modulates gene expression, we predicted that miR-34a formed complementary base pairing with NEAT1 using the online software program StarBase v2.0 [30]. A dual-luciferase reporter assay identified that NEAT1 contains a binding site for miR-34a (Figure 4(a)). Next, NEAT1 knockdown significantly ameliorated downregulation of miR-34a induced by TGF-*β*2 (Figure 4(b)). Next, the NEAT1 levels were increased by transfecting with the pcDNA3.1-NEAT1 vector or mut vector in primary HLECs (Figure 4(c)). Furthermore, miR-34a levels were downregulated in primary LECs treated with the pcDNA3.1-NEAT1 vector (Figure 4(d)). However, overexpression of NEAT1 using the pcDNA3.1-NEAT1-mut vector (mutations in the miRNA-34a response elements) did not affect the expression of miR-34a (Figure 4(d)). In addition, NEAT1 levels were unchanged after overexpression or knockdown of miR-34a in primary LECs (Figure 4(e)).

Growing evidence indicated that Ago2 plays a key role in catalytic activity during the silencing processes of RNA-induced silencing complex (RISC) [31]. To explore whether NEAT1 regulated miR-34a in an Ago2-dependent manner, we performed an anti-Ago2 RIP assay on HLECs. The endogenous NEAT1 pulldown was increased in HLECs which were transiently transfected with overexpression of miR-34a and decreased after knockdown of miR-34a (Figure 4(f)). Overall, we confirmed that NEAT1 negatively regulated miR-34a levels through “sponging” miR-34a.

### 3.5. Knockdown of NEAT1 Inhibits Snail1, a Target of miR-34a

**Figure 5:**
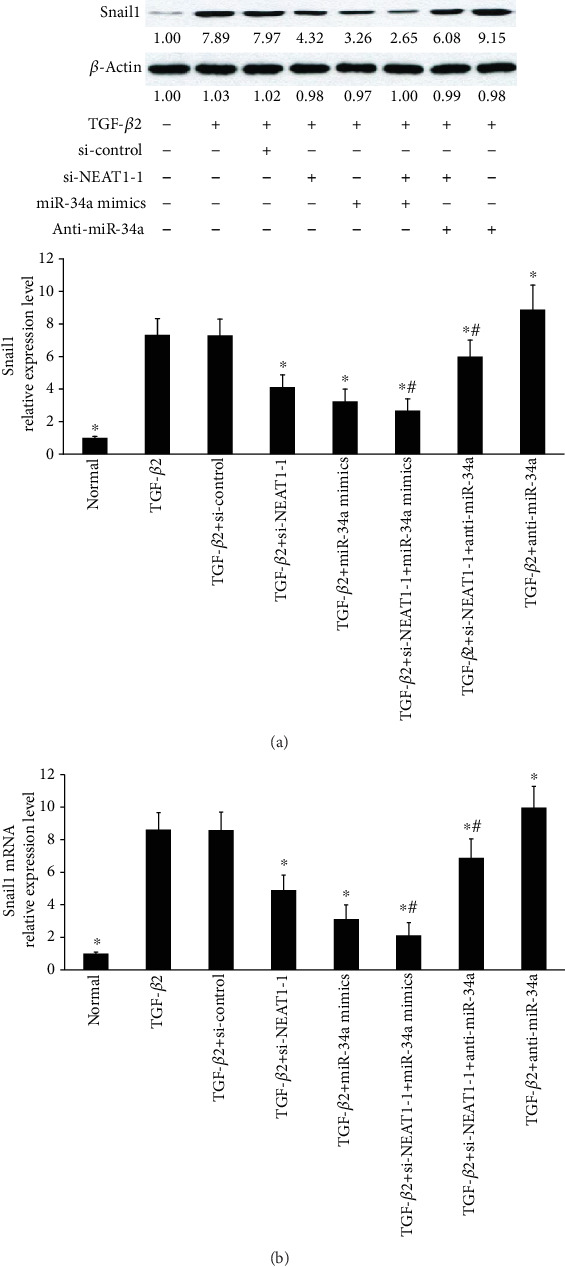
Knockdown of NEAT1 inhibits Snail1, a target of miR-34a. (a) The Snail1 protein levels were determined by Western blot analysis. The primary HLECs were treated with TGF-*β*2 (5 ng/ml) for 48 h before incubation with NEAT1 siRNAs for 24 h or anti-miR-34a for 6 h or miR-34a mimics for 6 h. (b) Snail1 mRNA was detected by qRT-PCR after overexpression or knockdown of miR-34a or knockdown of NEAT1. ^∗^*P* < 0.05 compared with the TGF-*β*2 group. ^#^*P* < 0.05 compared with the TGF-*β*2+si-NEAT1-1 group. The error bars represent the mean ± SE of six independent experiments.

Next, we investigated whether NEAT1 was involved in Snail1 expression induced by TGF-*β*2 through directly controlling miR-34a. NEAT1 knockdown suppressed the Snail1 protein and mRNA levels induced by TGF-*β*2 in primary HLECs (Figures 5(a) and 5(b)). Overexpression of miR-34a enhanced these effects. However, miR-34a knockdown ameliorated these effects (Figures 5(a) and 5(b)). These data suggest that NEAT1 partially controls Snail1 levels induced by TGF-*β*2 through competing with miRNA-34a.

### 3.6. Zeb1 Is a Target of miR-204 in Primary HLECs

**Figure 6:**
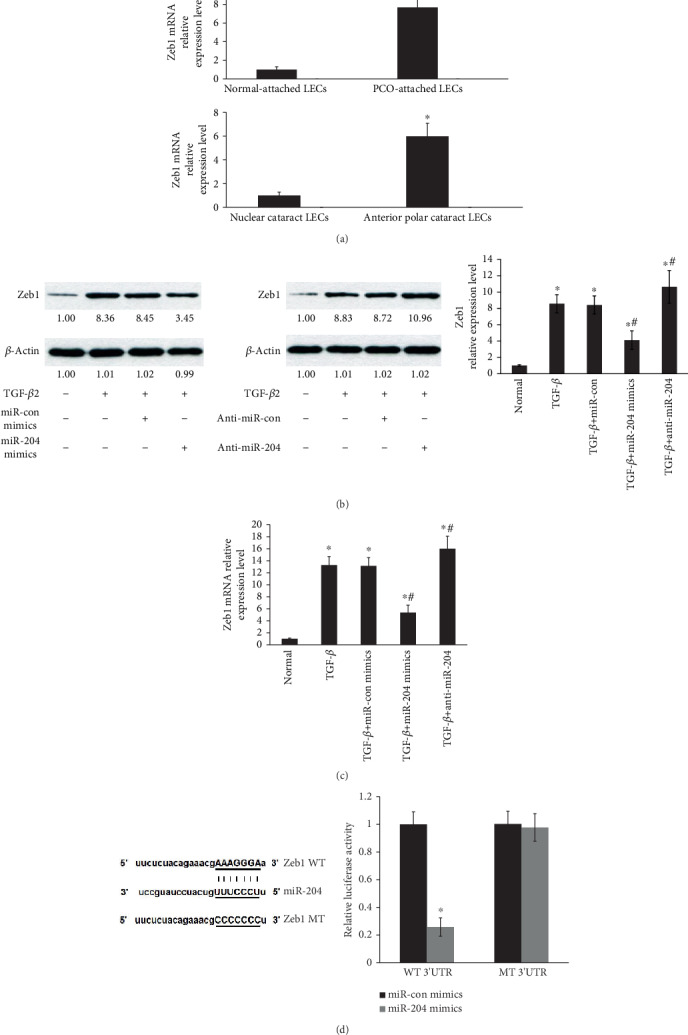
Zeb1 is a target of miR-204 in primary HLECs. (a) Zeb1 mRNA was determined by qRT-PCR. ^∗^*P* < 0.05 compared with normal-attached LECs or nuclear cataracts. (b) Zeb1 protein levels in primary HLECs were determined by Western blot analysis after overexpression or knockdown of miR-204. (c) Zeb1 mRNA expression was determined by qRT-PCR after overexpression or knockdown of miR-204. ^∗^*P* < 0.05 compared with the normal or TGF-*β*2 group. (b, c) ^∗^*P* < 0.05 compared with the normal group. ^#^*P* < 0.05 compared with the group with TGF-*β*2. (d) The luciferase reporter assays indicated that miR-204 directly targets Zeb1 in LECs. ^∗^*P* < 0.05 compared with the miR-204 control group. The error bars represent the mean ± SE of six independent experiments.

We hypothesized that miR-204 can inhibit Zeb1 translation via TargetScan (http://www.targetscan.org/vert_72/) [28, 29]. Zeb1, known as a zinc finger transcription, plays an active role in the EMT process induced by TGF-*β* [32–34]. To confirm these, Zeb1 mRNA levels were determined by qRT-PCR (Figure 6(a)). Zeb1 was elevated by nearly 8-fold in human PCO-attached LECs compared with normal-attached LECs (Figure 6(a)). There was more Zeb1 expression in LECs obtained from ASC (Figure 6(a)). The data identified that Zeb1 is involved in the pathogenesis of PCO. Next, overexpression of miR-204 inhibited Zeb1 protein levels induced by TGF-*β*2 (Figure 6(b)). Additionally, knockdown of miR-204 resulted in upregulation of Zeb1 protein levels (Figure 6(b)). Consistent with these results, qRT-PCR showed that miR-204 overexpression suppressed Zeb1 mRNA induced by TGF-*β*2 and miR-204 knockdown increased Zeb1 expression (Figure 6(c)). Finally, the “AAAGGGA” which is the 3′UTR of Zeb1 mRNA containing the predicted miR-204 binding sites changed into “CCCCCCC”, which was defined as MT 3′UTR. We identified that miR-204 directly targets Zeb1 in LECs by luciferase reporter assays (Figure 6(d)).

### 3.7. Zeb1 Negatively Regulated miR-204 Levels

**Figure 7:**
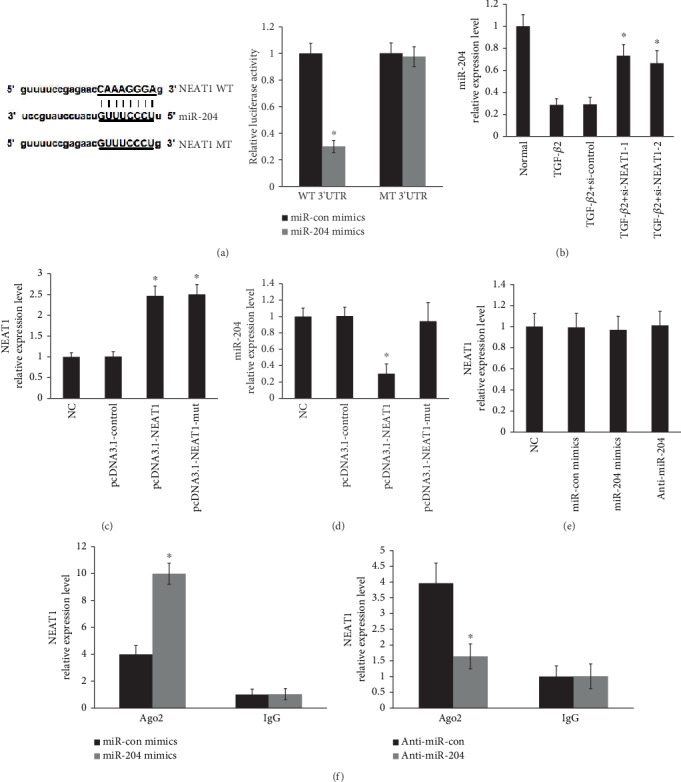
NEAT1 negatively regulated miR-204 levels. (a) NEAT1 contained a binding site for miR-204 in HLECs by the luciferase reporter assays. ^∗^*P* < 0.05 compared with the miR-204 control group. (b) miR-204 expression was determined by qRT-PCR. ^∗^*P* < 0.05 compared with the normal and TGF-*β*2 groups. (c) NEAT1 expression was determined by qRT-PCR. ^∗^*P* < 0.05 compared with the normal and pcDNA3.1-control groups. (d) miR-204 expression was determined by qRT-PCR. ^∗^*P* < 0.05 compared with the pcDNA3.1-NEAT1-mut group. (e) NEAT1 expression was determined by qRT-PCR. (f) NEAT1 expression was determined by qRT-PCR. ^∗^*P* < 0.05 compared with the miRNA control group. The error bars represent the mean ± SE of six independent experiments.

A dual-luciferase reporter assay indicated that NEAT1 contains a binding site for miR-204 (Figure 7(a)). NEAT1 knockdown significantly attenuated downregulation of miR-204 by TGF-*β*2 (Figure 7(b)). NEAT1 expression was elevated by transfecting with the pcDNA3.1-NEAT1 vector or mut vector in primary HLECs (Figure 7(c)). Moreover, miR-204 levels were suppressed in primary LECs treated with the pcDNA3.1-NEAT1 vector (Figure 7(d)). However, NEAT1 overexpression using the pcDNA3.1-NEAT1-mut vector (mutations in the miRNA-204 response elements) did not affect miR-204 expression (Figure 7(d)). Additionally, overexpression or knockdown of miR-204 did not change NEAT1 expression in primary LECs (Figure 7(e)). An anti-Ago2 RIP assay showed that overexpression of miR-204 resulted in upregulation of the endogenous NEAT1 pulldown and knockdown of miR-204 inhibited the endogenous NEAT1 pulldown (Figure 7(f)). These data suggest that NEAT1 negatively regulated miR-204 levels through “sponging” miR-204.

### 3.8. Knockdown of NEAT1 Inhibits Zeb1, a Target of miR-204

**Figure 8:**
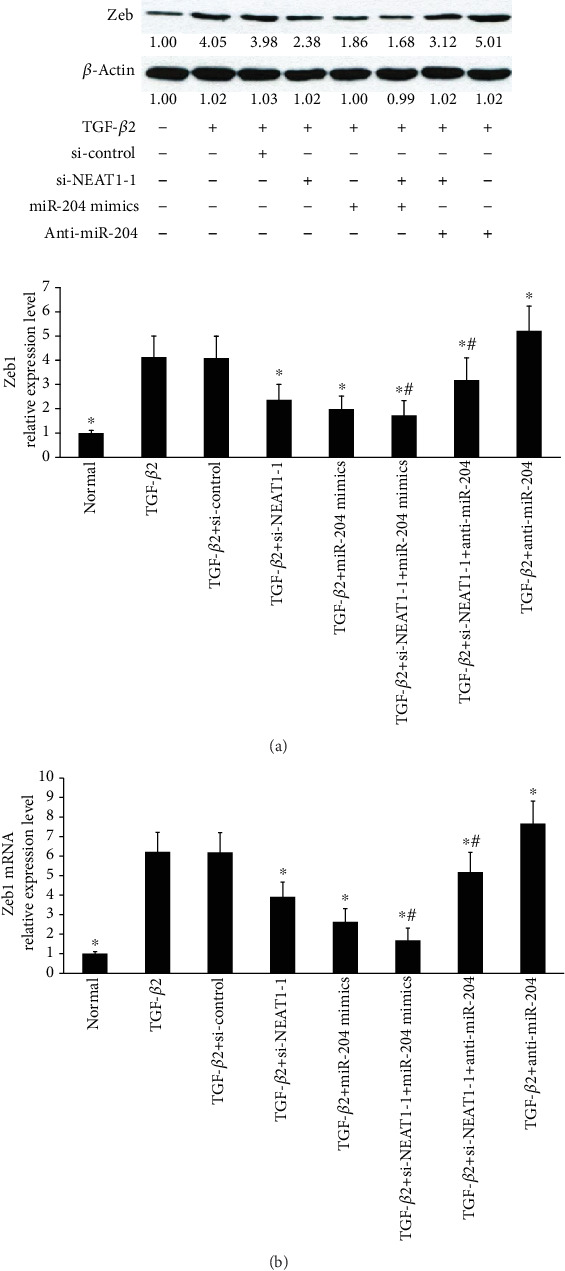
NEAT1 knockdown inhibits Zeb1, a target of miR-204. (a) Zeb1 protein levels were determined by Western blot analysis in the primary HLECs. LECs were treated with TGF-*β*2 (5 ng/ml) for 48 h before incubation with NEAT1 siRNAs for 24 h or anti-miR-204 for 6 h or miR-204 mimics for 6 h. (b) Zeb1 mRNA levels were determined by qRT-PCR after overexpression or knockdown of miR-204 or knockdown of NEAT1. ^∗^*P* < 0.05 compared with the TGF-*β*2 group. ^#^*P* < 0.05 compared with the TGF-*β*2+si-NEAT1-1 group. The error bars represent the mean ± SE of six independent experiments.

NEAT1 knockdown inhibited Zeb1 protein and mRNA expression induced by TGF-*β*2 in primary HLECs (Figures 8(a) and 8(b)). miR-204 overexpression enhanced these effects. However, knockdown of miR-204 attenuated these effects (Figures 8(a) and 8(b)). Collectively, these data indicated that NEAT1 partially controls Zeb1 expression induced by TGF-*β*2 through competing with miRNA-204.

### 3.9. NEAT1/Snail1 and NEAT1/Zeb1 Pathways Are Involved in TGF-*β*2-Induced EMT of LECs

**Figure 9:**
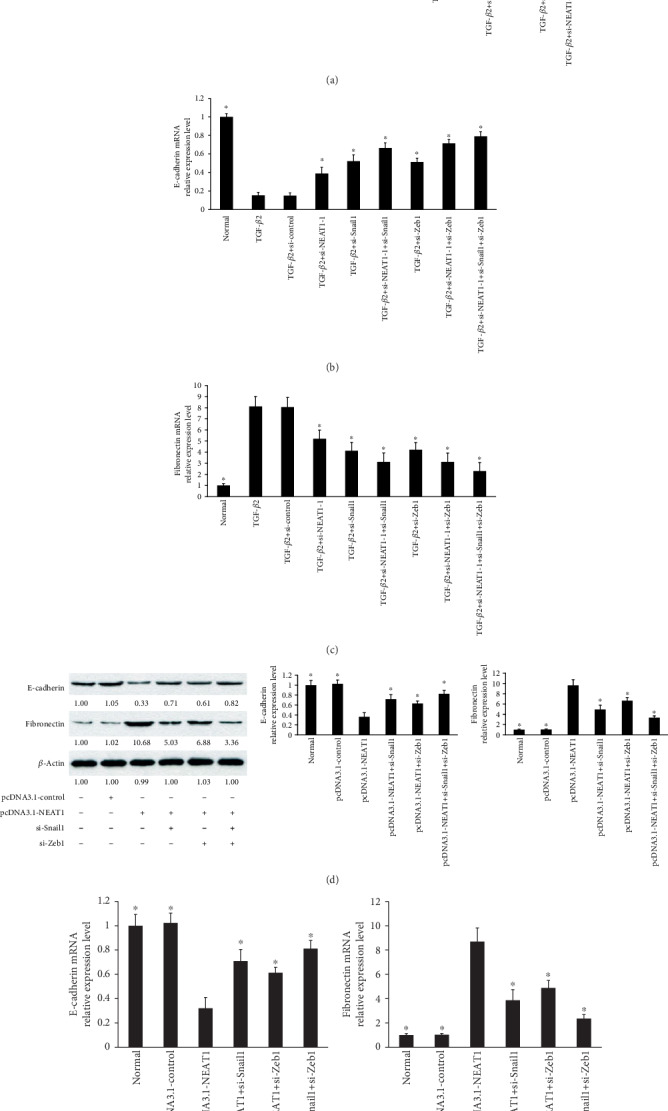
NEAT1/Snail1 and NEAT1/Zeb1 pathways are involved in TGF-*β*2-induced EMT of LECs. (a) E-cadherin and fibronectin protein levels in primary HLECs were determined by Western blot analysis after knockdown of NEAT1, Snail1, and Zeb1. (b) E-cadherin mRNA levels were determined by qRT-PCR in primary HLECs after knockdown of NEAT1, Snail1, and Zeb1. (c) Fibronectin mRNA expression was determined by qRT-PCR in primary HLECs after knockdown of NEAT1, Snail1, and Zeb1. (b, c) ^∗^*P* < 0.05 compared with the TGF-*β*2 group. (d) E-cadherin and fibronectin protein levels in primary HLECs were determined by Western blot analysis after overexpression of NEAT1 or knockdown of Snail1 or Zeb1. (e) E-cadherin mRNA levels were determined by qRT-PCR in primary HLECs after overexpression of NEAT1 or knockdown of Snail1 or Zeb1. (f) Fibronectin mRNA was determined by qRT-PCR in primary HLECs after overexpression of NEAT1 or knockdown of Snail1 or Zeb1. (e, f) ^∗^*P* < 0.05 compared with the pcDNA3.1-NEAT1 group. The error bars represent the mean ± SE of six independent experiments.

The above findings, which suggest that NEAT1 acts as a ceRNA targeting Snail1 by binding miR-34a and Zeb1 via binding miR-204, prompted us to explore whether the NEAT1/Snail1 and NEAT1/Zeb1 pathways are involved in TGF-*β*2-induced EMT of HLECs. To demonstrate it, we first evaluated the protein levels of E-cadherin and fibronectin by Western blot analysis. TGF-*β*2-induced fibronectin was inhibited by knockdown of NEAT1, Snail1, and Zeb1 in primary HLECs (Figure 9(a)). Next, TGF-*β*2 suppressed the levels of E-cadherin in primary HLECs, but the tendency was reversed by NEAT1, Snail1, and Zeb1 knockdown (Figure 9(a)). As expected, these effects were enhanced by NEAT1 knockdown together with Snail1 and Zeb1 knockdown simultaneously. Additionally, qRT-PCR also showed similar effects (Figures 9(b) and 9(c)).

Furthermore, the protein and mRNA expression of E-cadherin was inhibited by treatment with the pcDNA3.1-NEAT1 vector, but the tendency was reversed by Snail1 and Zeb1 knockdown, respectively (Figures 9(d) and 9(e)). As expected, these effects were amplified by Snail1 knockdown together with Zeb1 knockdown simultaneously (Figures 9(d) and 9(e)). In addition, overexpression of NEAT1 resulted in the increase of protein and mRNA expression of fibronectin, but these effects were suppressed using Snail1 siRNA and Zeb1 siRNA, respectively (Figures 9(d) and 9(f)). As expected, the tendency was enhanced by Snail1 knockdown together with Zeb1 knockdown simultaneously (Figures 9(d) and 9(e)). Overall, these data indicated that the NEAT1/Snail1 and NEAT1/Zeb1 pathways are involved in TGF-*β*2-induced EMT of HLECs.

## 4. Discussion

Improved artificial IOL design has restricted and inhibited the progression of PCO to some degree, but PCO remains a common complication of cataract surgery [3, 4]. Various cellular processes and signaling molecules are involved in PCO; however, the pathogenic mechanism of PCO is still unknown [3, 4]. In this study, our data clearly indicated that TGF-*β*2 induces EMT in primary HLECs through a NEAT1-dependent mechanism. NEAT1 overexpression inhibited miR-34a and miR-204 expression in primary HLECs using a Human miRNA Microarray System. Mechanistic studies revealed that NEAT1 negatively regulates miR-34a or miR-204, and miR-34a or miR-204 directly targets Snail1 or Zeb1, respectively. Finally, our results identified that the NEAT1/miR-34a/Snail1 and NEAT1/miR-204/Zeb1 pathways are involved in TGF-*β*2-induced EMT of HLECs.

Although lncRNA NEAT1 is without functional protein-coding ability and cannot translate into functional proteins, NEAT1 can drive several transcriptional and posttranscriptional processes [35]. NEAT1 was found to be involved in the regulation of cell growth, migration, and stem cell-like phenotype [35]. Specifically, increasing evidence suggested that NEAT1 can promote EMT [36–38]. For example, higher expression levels of NEAT1 were positively correlated with prognosis of breast cancer (BC) patients and NEAT1 knockdown suppressed N-cadherin expression while E-cadherin was upregulated [36]. Another group also found that the expression of NEAT1 was significantly increased in hepatocellular carcinoma (HCC) tissues and NEAT1 promotes tumor cell EMT, migration, and invasion capacities [37]. Others have reported that overexpression of NEAT1 is corrected with clinical stage, distant metastasis, and prognosis of gastric cancer and knockdown of NEAT1 suppressed EMT-associated protein expression of gastric cancer cell [38]. In the current study, we also uncovered that TGF-*β*2 induces EMT through a NEAT1-dependent mechanism and NEAT1 promotes EMT in primary HLECs.

On the other hand, increased evidence reveals that NEAT1 could abolish miRNA-mediated suppression of their target genes by sponging a set of miRNAs, such as miR-107, miR-193a, and miR-218 [35]. In particular, the previous studies have showed that NEAT1 could negatively regulate miR-34a or miR-204 through “sponging” miR-34a or miR-204, respectively [39, 40]. For example, NEAT1 could promote renal cell carcinoma (RCC) progression through the miR-34a/c-Met axis [39]. Moreover, NEAT1 could regulate the proliferation, migration, and apoptosis of human retinoblastoma cells via regulation of the miR-204/CXCR4 axis [40]. Interestingly, in the current data, we also found that a total of 216 miRNAs exhibited significant differential expression, and the expression of miR-34a and miR-204 which are the top downregulated miRNAs is decreased by nearly 6-fold in the NEAT1 overexpression HLECs. We further demonstrated that miR-34a and miR-204 expression was downregulated by nearly 5-fold in human PCO-attached LECs, and miR-34a and miR-204 are involved in the pathogenesis of PCO. We also further confirmed NEAT1 could negatively regulate miR-34a or miR-204 through “sponging” miR-34a or miR-204 in the LECs by RIP study and luciferase assay, respectively.

Lately, many transcriptional repressors of E-cadherin have been confirmed, and these included Snail1 which is from basic helix-loop-helix (bHLH) families and double zinc finger E-box binding (ZEB) transcription factors [41]. Furthermore, Snail1 and Zeb1 are typically upregulated induced by TGF-*β* in EMT [25, 26, 32–34]. In the current study, Snail1 and Zeb1 levels were elevated in human PCO-attached LECs compared with normal-attached LECs. Our findings identified that Snail1 and Zeb1 are involved in the pathogenesis of PCO. Additionally, it is known that TGF-*β*2-induced EMT plays a pivotal role in PCO progression [7, 8, 42]. We found that NEAT1 knockdown suppressed Snail1 expression induced by TGF-*β*2 and miR-34a overexpression enhanced these effects in primary HLECs. Moreover, knockdown of NEAT1 resulted in downregulation of Zeb1 induced by TGF-*β*2 and miR-204 knockdown attenuated these effects. These findings suggested that NEAT1 controls Snail1 and Zeb1 expression induced by TGF-*β*2 through competing with miR-34a and miRNA-204. The mechanism is that miR-34a directly targets Snail1 and miR-204 directly targets Zeb1 in LECs confirmed by the luciferase reporter assays. Overall, the current findings indicated TGF-*β*2 induces downregulation of epithelial differentiation markers (i.e., E-cadherin) and upregulation of mesenchymal cell markers (i.e., fibronectin) in primary HLECs through the NEAT1/miR-34a/Snail1 and NEAT1/miR-204/Zeb1 pathways.

## 5. Conclusion

In summary, the current study provided the evidence that TGF-*β*2 induces EMT via a NEAT1-dependent mechanism in primary HLECs. Mechanistic studies revealed that NEAT1 negatively regulates miR-34a or miR-204 through “sponging” miR-34a or miR-204, respectively, and in turn induces Snail1 or Zeb1. Thus, these findings also identified that the NEAT1/miR-34a/Snail1 and NEAT1/miR-204/Zeb1 pathways are involved in TGF-*β*2-induced EMT of LECs, and NEAT1 is a potential therapeutic target for the treatment of PCO.

## Figures and Tables

**Table 1:** The sequences used for NEAT1 siRNA.

	Sequence	
	Sense	Antisense
siNEAT1-1	5′-GAUGCUGCAUCUUCUAAAUTT-3′	5′-AUUUAGAAGAUGCAGCAUCTT-3′
siNEAT1-2	5′-GCAGGUUGAAGGGAAUUCUTT-3′	5′-AGAAUUCCCUUCAACCUGCTT-3′
si-control	5′-UUCUCCGAACGUGUCACGUTT-3′	5′-ACGUGACACGUUCGGAGAATT-3′

**Table 2:** Primers used for qRT-PCR.

Primers	Sequence	
	Sense	Antisense
GAPDH	5′-AGGTCGGTGTGAACGGATTTG-3′	5′-TGTAGACCATGTAGTTGAGGTCA-3′
U6	5′-CTCGCTTCGGCAGCACA-3′	5′-AACGCTTCACGAATTTGCGT-3′
miR-34a	5′-TGGCAGTGTCTTAGCTGGTTGT-3′	5′-GCGAGCACAGAATTAATACGAC-3′
miR-204	5′-TGGTTTTTTTTTAAATTAAGTTAGTAAAGT-3′	5′-ACAACCTACACAAAACAACCTATAATC-3′
NEAT1	5′-TTTGTGCTTGGAACCTTGCT-3′	5′-TCAACGCCCCAAGTTATTTC-3′
E-cadherin	5′-CGAGAGCTACACGTTCACGG-3′	5′-GGGTGTCGAGGGAAAAATAGG-3′
Fibronectin	5′-TCTGTGCCTCCTATCTATGTGC-3′	5′-GAGGGACCACGACAACTCTTC-3′
Snail1	5′-CTGCGGGAAGGCCTTCTCT-3′	5′-CGCCTGGCACTGGTACTTCTT-3′
Zeb1	5′-ACTGTTTGTAGCGACTGGATT-3′	5′-TAAAGTGGCGGTAGATGGTA-3′

## Data Availability

All relevant data used to support the findings of this study are included within the article.

## Supplementary Materials

Supplementary Materials Figure S1: TGF-*β*2 induces downregulation of E-cadherin and upregulation of fibronectin in primary HLECs through a NEAT1-dependent mechanism. Immunocytofluorescence showed that the expression of E-cadherin (green) and fibronectin (green) and the morphology of primary HLECs. The primary HLECs were treated with TGF-*β*2 (5 ng/ml) for 48 h before incubation with 100 nM siNEAT1-1 or siNEAT1-2 for 24 h.

## References

[1] Lee C. M., Afshari N. A. (2017). The global state of cataract blindness. *Current Opinion in Ophthalmology*.

[2] Yan W., Wang W., van Wijngaarden P., Mueller A., He M. (2018). Longitudinal changes in global cataract surgery rate inequality and associations with socioeconomic indices. *Clinical & Experimental Ophthalmology*.

[3] Wormstone I. M., Eldred J. A. (2016). Experimental models for posterior capsule opacification research. *Experimental Eye Research*.

[4] Wormstone I. M., Wang L., Liu C. S. C. (2009). Posterior capsule opacification. *Experimental Eye Research*.

[5] Dong N., Xu B., Xu J. (2018). EGF-mediated overexpression of Myc attenuates miR-26b by recruiting HDAC3 to induce epithelial-mesenchymal transition of lens epithelial cells. *BioMed Research International*.

[6] Dong N. (2019). Long noncoding RNA MALAT1 acts as a competing endogenous RNA to regulate TGF-*β*2 induced epithelial-mesenchymal transition of lens epithelial cells by a microRNA-26a-dependent mechanism. *BioMed Research International*.

[7] Smith A. J. O., Eldred J. A., Wormstone I. M. (2019). Resveratrol inhibits wound healing and lens fibrosis: a putative candidate for posterior capsule opacification prevention. *Investigative Ophthalmology & Visual Science*.

[8] Srinivasan Y., Lovicu F. J., Overbeek P. A. (1998). Lens-specific expression of transforming growth factor beta1 in transgenic mice causes anterior subcapsular cataracts. *The Journal of Clinical Investigation*.

[9] Saika S., Miyamoto T., Kawashima Y. (2000). Immunolocalization of TGF-beta1, -beta2, and -beta3, and TGF-beta receptors in human lens capsules with lens implants. *Graefe's Archive for Clinical and Experimental Ophthalmology*.

[10] Gordon-Thomson C., de Iongh R. U., Hales A. M., Chamberlain C. G., McAvoy J. W. (1998). Differential cataractogenic potency of TGF-beta1, -beta2, and -beta3 and their expression in the postnatal rat eye. *Investigative Ophthalmology & Visual Science*.

[11] Gugnoni M., Ciarrocchi A. (2019). Long noncoding RNA and epithelial mesenchymal transition in cancer. *International Journal of Molecular Sciences*.

[12] Xu Q., Deng F., Qin Y. (2016). Long non-coding RNA regulation of epithelial-mesenchymal transition in cancer metastasis. *Cell death & disease,*.

[13] Zhang B., Chen Y., Qiu M., Ding Z. (2017). Long noncoding RNA expression profile in HLE B-3 cells during TGF-*β*2-induced epithelial-mesenchymal transition. *BMC Ophthalmology*.

[14] Chen B., Ma J., Li C., Wang Y. (2018). Long noncoding RNA KCNQ1OT1 promotes proliferation and epithelial mesenchymal transition by regulation of SMAD4 expression in lens epithelial cells. *Molecular Medicine Reports*.

[15] Li X., Wang F., Ren M., Du M., Zhou J. (2019). The effects of c-Src kinase on EMT signaling pathway in human lens epithelial cells associated with lens diseases. *BMC Ophthalmology*.

[16] Wang X., Wang B., Zhao N. (2019). Pharmacological targeting of BET bromodomains inhibits lens fibrosis via downregulation ofMYCExpression. *Investigative Ophthalmology & Visual Science*.

[17] Dong N., Tang X., Xu B. (2015). MiRNA-181a inhibits the proliferation, migration, and epithelial–mesenchymal transition of lens epithelial cells. *Investigative Ophthalmology & Visual Science*.

[18] Dong N., Xu B., Benya S. R., Tang X. (2014). MiRNA-26b inhibits the proliferation, migration, and epithelial-mesenchymal transition of lens epithelial cells. *Molecular and Cellular Biochemistry*.

[19] Dong N., Xu B., Shi H. (2018). Long noncoding RNA MALAT1 acts as a competing endogenous RNA to regulate Amadori-glycated albumin-induced MCP-1 expression in retinal microglia by a microRNA-124-dependent mechanism. *Inflammation Research*.

[20] Cesana M., Cacchiarelli D., Legnini I. (2011). A long noncoding RNA controls muscle differentiation by functioning as a competing endogenous RNA. *Cell*.

[21] Hansen T. B., Jensen T. I., Clausen B. H. (2013). Natural RNA circles function as efficient microRNA sponges. *Nature*.

[22] Feng D., Zhu N., Yu C., Lou D. (2019). MicroRNA-34a suppresses human lens epithelial cell proliferation and migration via downregulation of c-Met. *Clinica Chimica Acta*.

[23] Han R., Hao P., Wang L. (2019). MicroRNA-34a inhibits epithelial-mesenchymal transition of lens epithelial cells by targeting Notch1. *Experimental Eye Research*.

[24] Wang Y., Li W., Zang X. (2013). MicroRNA-204-5p regulates epithelial-to-mesenchymal transition during human posterior capsule opacification by targeting SMAD4. *Investigative Ophthalmology & Visual Science*.

[25] Parapuram S. K., Chang B., Li L. (2009). Differential effects of TGF*β* and vitreous on the transformation of retinal pigment epithelial cells. *Investigative Ophthalmology & Visual Science*.

[26] De Craene B., van Roy F., Berx G. (2005). Unraveling signalling cascades for the Snail family of transcription factors. *Cellular Signalling*.

[27] Liu L., Xiao W. (2017). Notch1 signaling induces epithelial-mesenchymal transition in lens epithelium cells during hypoxia. *BMC Ophthalmology*.

[28] Dong N., Xu B., Shi H., Lu Y. (2016). miR-124 regulates Amadori-Glycated albumin-induced retinal microglial activation and Inflammation by targeting Rac1. *Investigative Ophthalmology & Visual Science*.

[29] Dong N., Xu B., Shi H., Tang X. (2015). Baicalein inhibits amadori-glycated albumin-induced mcp-1 expression in retinal ganglion cells via a microrna-124-dependent mechanism. *Investigative Ophthalmology & Visual Science*.

[30] Li J. H., Liu S., Zhou H., Qu L. H., Yang J. H. (2013). starBase v2.0: decoding miRNA-ceRNA, miRNA-ncRNA and protein-RNA interaction networks from large-scale CLIP-Seq data. *Nucleic Acids Research*.

[31] Gaiti F., Degnan B. M., Tanurdžić M. (2018). Long non-coding regulatory RNAs in sponges and insights into the origin of animal multicellularity. *RNA Biology*.

[32] Gao J., Zhu Y., Nilsson M., Sundfeldt K. (2014). TGF-*β* isoforms induce EMT independent migration of ovarian cancer cells. *Cancer Cell International*.

[33] Miyazono K. (2009). Transforming growth factor-beta signaling in epithelial-mesenchymal transition and progression of cancer. *Proceedings of the Japan Academy Series B, Physical and Biological Sciences*.

[34] Li C., Zheng H., Hou W. (2019). Long non-coding RNA linc00645 promotes TGF-*β*-induced epithelial-mesenchymal transition by regulating miR-205-3p-ZEB1 axis in glioma. *Cell Death & Disease*.

[35] Dong P., Xiong Y., Yue J. (2018). Long non-coding RNA NEAT1: a novel target for diagnosis and therapy in human tumors. *Frontiers in Genetics*.

[36] Zhang M., Wu W. B., Wang Z. W., Wang X. H. (2017). lncRNA NEAT1 is closely related with progression of breast cancer via promoting proliferation and EMT. *European Review for Medical and Pharmacological Sciences*.

[37] Zheng X., Zhang Y., Liu Y. (2018). HIF-2*α* activated lncRNA NEAT1 promotes hepatocellular carcinoma cell invasion and metastasis by affecting the epithelial-mesenchymal transition. *Journal of Cellular Biochemistry*.

[38] Fu J. W., Kong Y., Sun X. (2016). Long noncoding RNA NEAT1 is an unfavorable prognostic factor and regulates migration and invasion in gastric cancer. *Journal of Cancer Research and Clinical Oncology*.

[39] Liu F., Chen N., Gong Y., Xiao R., Wang W., Pan Z. (2017). The long non-coding RNA NEAT1 enhances epithelial-to-mesenchymal transition and chemoresistance via the miR-34a/c-Met axis in renal cell carcinoma. *Oncotarget*.

[40] Zhong W., Yang J., Li M., Li L., Li A. (2018). Long noncoding RNA NEAT1 promotes the growth of human retinoblastoma cells via regulation of miR-204/CXCR4 axis. *Journal of Cellular Physiology*.

[41] Imani S., Hosseinifard H., Cheng J., Wei C., Fu J. (2016). Prognostic value of EMT-inducing transcription factors (EMT-TFs) in metastatic breast cancer: a systematic review and meta-analysis. *Scientific Reports*.

[42] Zhang Z., Zhu H., Liu Y., Quan F., Zhang X., Yu L. (2018). LncRNA HOTAIR mediates TGF-*β*2-induced cell growth and epithelial-mesenchymal transition in human lens epithelial cells. *Acta Biochimica et Biophysica Sinica*.

