# Supplementary material for: Long Noncoding RNA NEAT1 Regulates TGF-β2-Induced Epithelial-Mesenchymal Transition of Lens Epithelial Cells through the miR-34a/Snail1 and miR-204/Zeb1 Pathways
Source: Biomed Res Int. 2020 May 31;2020:8352579. doi: 10.1155/2020/8352579 (PMC7284955; doi:10.1155/2020/8352579)
Supplement: Supplementary Materials — Figure S1: TGF-β2 induces downregulation of E-cadherin and upregulation of fibronectin in primary HLECs through a NEAT1-dependent mechanism. Immunocytofluorescence showed that the expression of E-cadherin (green) and fibronectin (green) and the morphology of primary HLECs. The primary HLECs were treated with TGF-β2 (5 ng/ml) for 48 h before incubation with 100 nM siNEAT1-1 or siNEAT1-2 for 24 h. [file 8352579.f1.pdf]

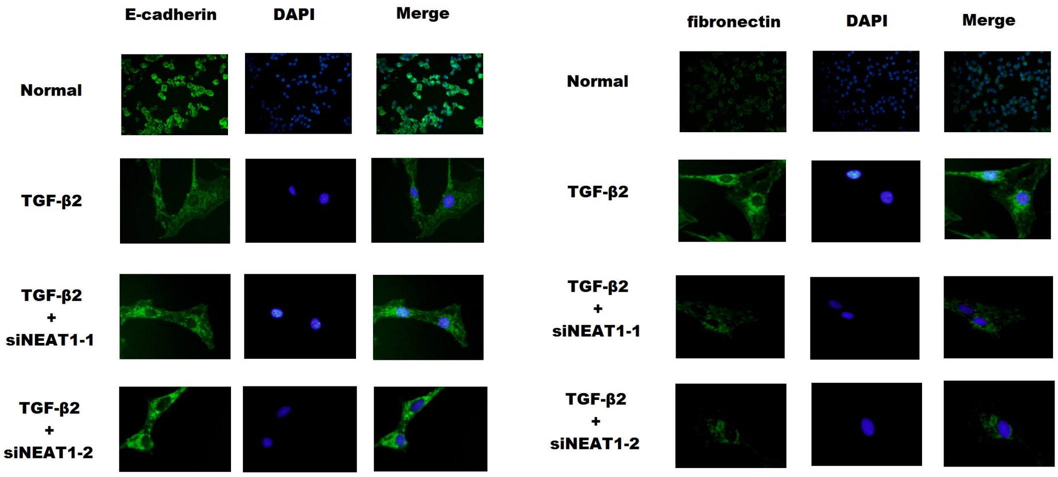

Supplementary Figure S1: TGF-β2 induces down-regulation of E-cadherin and up-regulation of fibronectin in primary HLECs through a NEAT1-dependent mechanism. Immunocytofluorescence showed that the expression of E-cadherin (green) and fibronectin (green) and the morphology of primary HLECs. The primary HLECs were treated with TGF-β2 (5 ng/ml) for 48 h before incubation with 100 nM siNEAT1-1 or siNEAT1-2 for 24 h.
